# Liver resection versus liver transplantation for hepatocellular carcinoma within the Milan criteria based on estimated microvascular invasion risks

**DOI:** 10.1093/gastro/goad035

**Published:** 2023-06-26

**Authors:** Pinghua Yang, Fei Teng, Shilei Bai, Yong Xia, Zhihao Xie, Zhangjun Cheng, Jun Li, Zhengqing Lei, Kui Wang, Baohua Zhang, Tian Yang, Xuying Wan, Hao Yin, Hao Shen, Timothy M Pawlik, Wan Yee Lau, Zhiren Fu, Feng Shen

**Affiliations:** Department of Hepatic Surgery IV and Clinical Research Institute, The Eastern Hepatobiliary Surgery Hospital, Naval Medical University, Shanghai, P. R. China; Department of Liver Surgery and Organ Transplantation, The Changzheng Hospital, Naval Medical University, Shanghai, P. R. China; Department of Hepatic Surgery IV and Clinical Research Institute, The Eastern Hepatobiliary Surgery Hospital, Naval Medical University, Shanghai, P. R. China; Department of Hepatic Surgery IV and Clinical Research Institute, The Eastern Hepatobiliary Surgery Hospital, Naval Medical University, Shanghai, P. R. China; Department of Hepatic Surgery IV and Clinical Research Institute, The Eastern Hepatobiliary Surgery Hospital, Naval Medical University, Shanghai, P. R. China; Department of Hepatic Surgery IV and Clinical Research Institute, The Eastern Hepatobiliary Surgery Hospital, Naval Medical University, Shanghai, P. R. China; Department of General Surgery, The Affiliated Zhongda Hospital, Southeast University, Nanjing, Jiangsu, P. R. China; Department of Hepatic Surgery IV and Clinical Research Institute, The Eastern Hepatobiliary Surgery Hospital, Naval Medical University, Shanghai, P. R. China; Department of Hepatic Surgery IV and Clinical Research Institute, The Eastern Hepatobiliary Surgery Hospital, Naval Medical University, Shanghai, P. R. China; Department of General Surgery, The Affiliated Zhongda Hospital, Southeast University, Nanjing, Jiangsu, P. R. China; Department of Hepatic Surgery II and Clinical Research Institute, The Eastern Hepatobiliary Surgery Hospital, Naval Medical University, Shanghai, P. R. China; Department of Biliary Surgery IV and Clinical Research Institute, The Eastern Hepatobiliary Surgery Hospital, Naval Medical University, Shanghai, P. R. China; Department of Hepatic Surgery II and Clinical Research Institute, The Eastern Hepatobiliary Surgery Hospital, Naval Medical University, Shanghai, P. R. China; Department of Chinese Traditional Medicine, The Eastern Hepatobiliary Surgery Hospital, Naval Medical University, Shanghai, P. R. China; Department of Liver Surgery and Organ Transplantation, The Changzheng Hospital, Naval Medical University, Shanghai, P. R. China; Department of Hepatic Surgery IV and Clinical Research Institute, The Eastern Hepatobiliary Surgery Hospital, Naval Medical University, Shanghai, P. R. China; Department of Surgery, The Wexner Medical Center, Ohio State University, Columbus, OH, USA; Department of Hepatic Surgery IV and Clinical Research Institute, The Eastern Hepatobiliary Surgery Hospital, Naval Medical University, Shanghai, P. R. China; Faculty of Medicine, the Chinese University of Hong Kong, Prince of Wales Hospital, Shatin, New Territories, Hong Kong SAR, China; Department of Liver Surgery and Organ Transplantation, The Changzheng Hospital, Naval Medical University, Shanghai, P. R. China; Department of Hepatic Surgery IV and Clinical Research Institute, The Eastern Hepatobiliary Surgery Hospital, Naval Medical University, Shanghai, P. R. China

**Keywords:** hepatocellular carcinoma, microvascular invasion, liver resection, liver transplantation, prognosis

## Abstract

**Background:**

Preoperative prediction of microvascular invasion (MVI) in hepatocellular carcinoma (HCC) may optimize individualized treatment decision-making. This study aimed to investigate the prognostic differences between HCC patients undergoing liver resection (LR) and liver transplantation (LT) based on predicted MVI risks.

**Methods:**

We analysed 905 patients who underwent LR, including 524 who underwent anatomical resection (AR) and 117 who underwent LT for HCC within the Milan criteria using propensity score matching. A nomogram model was used to predict preoperative MVI risk.

**Results:**

The concordance indices of the nomogram for predicting MVI were 0.809 and 0.838 in patients undergoing LR and LT, respectively. Based on an optimal cut-off value of 200 points, the nomogram defined patients as high- or low-risk MVI groups. LT resulted in a lower 5-year recurrence rate and higher 5-year overall survival (OS) rate than LR among the high-risk patients (23.6% vs 73.2%, *P *<* *0.001; 87.8% vs 48.1%, *P *<* *0.001) and low-risk patients (19.0% vs 45.7%, *P *<* *0.001; 86.5% vs 70.0%, *P *=* *0.002). The hazard ratios (HRs) of LT vs LR for recurrence and OS were 0.18 (95% confidence interval [CI], 0.09–0.37) and 0.12 (95% CI, 0.04–0.37) among the high-risk patients and 0.37 (95% CI, 0.21–0.66) and 0.36 (95% CI, 0.17–0.78) among the low-risk patients. LT also provided a lower 5-year recurrence rate and higher 5-year OS rate than AR among the high-risk patients (24.8% vs 63.5%, *P *=* *0.001; 86.7% vs 65.7%, *P *=* *0.004), with HRs of LT vs AR for recurrence and OS being 0.24 (95% CI, 0.11–0.53) and 0.17 (95% CI, 0.06–0.52), respectively. The 5-year recurrence and OS rates between patients undergoing LT and AR were not significantly different in the low-risk patients (19.4% vs 28.3%, *P *=* *0.129; 85.7% vs 77.8%, *P *=* *0.161).

**Conclusions:**

LT was superior to LR for patients with HCC within the Milan criteria with a predicted high or low risk of MVI. No significant differences in prognosis were found between LT and AR in patients with a low risk of MVI.

## Introduction

Hepatocellular carcinoma (HCC) is one of the most common malignancies worldwide, with hepatitis B virus (HBV) infection accounting for 70%–90% of the cases of this malignancy [1]. Liver resection (LR) and liver transplantation (LT) may provide cures for patients with early-stage HCC [2, 3]. Previous studies comparing survival outcomes following LR and LT for patients with HCC within the Milan criteria reported comparable outcomes between the two procedures or favorable outcomes for LT [4–10]. These inconsistent results are likely associated with heterogeneity in the selection criteria for the two procedures and treatment protocols among different studies [4–12]. Additionally, many previous studies failed to consider the potential impact of the LR type (i.e. anatomical resection [AR] or non-anatomical resection [NAR]), which may have affected the comparative results [6, 8–10, 13]. Furthermore, the biological characteristics of HCC, which are typically manifested by the presence of microvascular invasion (MVI) [12, 14, 15] and are not included in the Milan criteria, may have affected the outcomes of HCC patients undergoing LR and LT.

The presence of MVI is associated with a significantly increased risk of tumor recurrence after LR or LT for HCC [14, 16]. Mazzaferro *et al.* [17] proposed the “up-to-seven rule” that includes the presence of MVI as an adverse and strong prognostic factor for HCC patients receiving LT. Other studies have suggested that the survival benefit of LT is reduced in patients with resectable HCC harboring MVI [12, 13]. Given that the diagnosis of MVI can only be obtained from post-operative histopathology on surgical specimens, using MVI to inform preoperative treatment decisions is difficult [3, 18]. As such, some studies have sought to predict the MVI risks based on the information available in the preoperative decision-making setting [19–23]. Identifying the prognostic differences between patients undergoing different treatment modalities for HCC based on preoperatively predicted MVI risks can provide evidence for using MVI in making treatment decisions.

The present study aimed to examine the relative long-term outcomes after LR and LT in patients with early-stage HCC who were technically suitable for both treatments based on the predicted risks of MVI.

## Materials and methods

### Study design

Consecutive patients who underwent LR or LT as first-line treatment for histopathologically confirmed HCC at the Eastern Hepatobiliary Surgery Hospital and Changzheng Hospital (Shanghai, China) were analysed. LR types included AR and NAR. Only patients with HCC who met the Milan criteria [23] were identified. Since the current study sought to compare the prognoses between patients undergoing LR and LT among those who were amenable to both treatments, LT patients with a Child–Pugh score beyond B7, clinically significant portal hypertension, refractory ascites, tumors located within 0.5 cm of the hepatic hilus, vena cava or the base of the main hepatic veins, and an inadequate volume of future liver remnant (FLR) were excluded because LR was not feasible or would be unsafe in such patients [2, 3]. Patients who underwent non-R0 resection were excluded [24]. In addition, both LT and LR patients who had a history of anticancer treatment for HCC or had received neoadjuvant or post-operative adjuvant therapy were excluded because these treatments might have affected the status of MVI or the impact of MVI on surgical prognosis [14, 25–27]. Patients with a history of other malignancies, no HBV infection, or missing clinicopathological data were excluded. This study was approved by the institutional ethics committee of each center. Informed consent was obtained from all patients before surgery for the use of their data for clinical research.

### Preoperative workup and surgery

Patients were routinely assessed for preoperative general performance and underwent laboratory examinations, including liver and renal function tests, coagulation profile, α-fetoprotein (AFP), carbohydrate antigen 19–9, carcinoembryonic antigen, HBV antigen/antibody, HBV deoxyribonucleic acid (HBV-DNA) level, and hepatitis C virus antibody. Other routine examinations included abdominal ultrasound, chest radiography, contrast-enhanced magnetic resonance imaging (MRI), and/or computed tomography (CT) of the abdomen and upper gastrointestinal endoscopy. Candidates for LT were also examined using chest CT, scintigraphy, positron emission tomography, and angiography. The clinical diagnosis of HCC was made according to the criteria of the American Association for the Study of Liver Diseases (AASLD) [3]. LR, which was based on the anatomical removal of one or more Couinaud segments containing the tumor together with the tumor-bearing portal vein and its corresponding hepatic territory, was classified as AR. Other resections that did not follow the hepatic segment anatomy were classified as NAR [28, 29]. The LT recipients underwent cadaveric donor LR, donor liver bench surgery, and recipient operations. Classic orthotopic LT without a venovenous bypass is the primary technique used for vena cava reconstruction. Duct-to-duct anastomosis was used for biliary reconstruction. Immunosuppressive therapy consisted of tacrolimus (FK506), mycophenolate mofetil (MMF), and prednisone. Post-operative histopathological studies were routinely performed. The Edmondson–Steiner grade was used to determine tumor differentiation [30]. R0 resection was defined as the complete removal of macroscopic nodules with a microscopic tumor-free resection margin [24]. The histopathological diagnosis of MVI was determined according to previously reported methods. Briefly, the presence of MVI was identified by the presence of cancer emboli or cancer cell nests in the small branches of the portal vein or hepatic vein in the adjacent liver tissues or in the large capsular vessels lined by endothelium, which can only be detected by using microscopy [15, 31–33]. All histopathological examinations were independently performed by three pathologists, who arrived at a consensus by discussion if any controversy existed.

### Estimation of preoperative MVI risk using a nomogram

Our group proposed a nomogram to predict MVI in patients with HCC within the Milan criteria using preoperative data, which was noted to have an optimal predictive performance [23]. The model incorporated tumor diameter, number, capsule status, AFP level, platelet count (PLT), HBV-DNA level, and a typical dynamic pattern of HCC on contrast-enhanced MRI [34]. Based on an optimal cut-off value of 200 nomogram points as determined by using receiver-operating characteristic curve analysis, patients who had nomogram points of ≥200 and ˂200 were classified as high- and low-risk MVI groups, respectively. At this optimal cut-off value, the model demonstrated good sensitivity, specificity, and positive and negative predictive values [23].

### Follow-up and end points

Patients were followed up for tumor recurrence surveillance once every 2–3 months within the first 2 years of surgery and then once every 4–6 months or earlier if clinically indicated. A contrast-enhanced CT scan or MRI of the abdomen and chest radiography or CT scan were performed once every 4–6 months or earlier if recurrence/metastasis was suspected. HCC recurrence was diagnosed using the AASLD criteria [3] and managed using multidisciplinary treatment [2, 3, 18]. For LT patients, the blood concentrations of immunosuppressive drugs and liver function were checked once every month within the first year and then once every 2–3 months. Drugs were adjusted according to the blood concentrations, liver function, and clinical manifestations.

The primary analysis of this study compared survival outcomes between patients undergoing LR and LT, and the subgroup analysis examined the prognostic differences between patients undergoing AR and LT. The end points of this study were overall survival (OS), defined as the interval from LR or LT to death from any cause or the last follow-up; and time to recurrence, defined as the interval between surgery and the first diagnosis of HCC recurrence.

### Statistical analysis

Variables are expressed as numbers (%) or means (standard deviations). Continuous variables were compared using the paired *t*-test or Mann–Whitney *U* test, as appropriate. Categorical variables were compared using the chi-squared test or Fisher’s exact test. The performance of the nomogram was measured using the concordance index (C-index), calculated using the rms package of R (version 3.0; http://www.r-project.org). The accuracy of the cut-off value of 200 nomogram points was assessed based on the sensitivity, specificity, and positive and negative predictive values [23]. Propensity score matching (PSM) was used to adjust for baseline differences between patients undergoing LT and LR. A propensity score was calculated using multivariable logistic regression and 3:1 matching between LR and LT patients [35]. Nearest neighbor and caliper matching without replacement were selected as the matching algorithm and the pairs on the propensity score logit were then matched within a range of 0.2 of a standard deviation. Among patients with a high risk of MVI, the variables used in PSM were hepatitis B e antigen (HBeAg), total bilirubin (TBIL), albumin (ALB), PLT, Child–Pugh grade, tumor number, and cirrhosis for comparing LR and LT; and TBIL, PLT, α fetoprotein (AFP), Child–Pugh grade, cirrhosis, and tumor number for comparing AR and LT. Among patients with a low risk of MVI, the variables were HBeAg, ALB, PLT, Child–Pugh grade, tumor number, and cirrhosis for comparing LR and LT; and HBeAg, ALB, Child–Pugh grade, cirrhosis, tumor diameter, and number for comparing AR and LT. Collinearity among the variables was assessed by examining the variance inflation factors; no collinearity was noted among the variables that were included in the statistical analysis. Survival curves were analysed using the Kaplan–Meier method and log-rank test. Cox proportional hazard regression models were used for the univariable and multivariable analyses of OS and recurrence. Preoperative variables potentially associated with prognosis and treatment type were used in the analysis. Variables with statistical significance in the univariable analysis as well as those usually reported to be associated with the prognosis of patients with HCC were included in the multivariable analysis. All reported *P-*values were two-sided and a *P-*value of <0.05 was considered statistically significant.

## Results

### Patient characteristics

Among the 2,455 patients who underwent LR between January 2010 and February 2015, 1,550 were excluded based on the eligibility criteria, and the remaining 905 patients were included in this study. Of these patients, 524 (57.9%) and 381 (42.1%) were treated with AR and NAR, respectively. Of the 496 patients treated with LT from January 2001 to February 2015, 117 were identified after excluding 379 patients who did not meet the eligibility criteria (Supplementary Figure 1). Some baseline differences were noted between the LT and LR patients (Supplementary Table 1). Compared with the LT patients, a higher proportion of the LR patients had HBeAg positivity (36.2% vs 21.3%), an ALB level of ≥3.5 g/dL (96.6% vs 88.0%), a PLT count of ≥100 × 10^9^/L (83.4% vs 70.1%), Child–Pugh grade A (97.9% vs 88.0%), and a solitary nodule (86.9% vs 62.4%) (all *P *≤* *0.001), whereas a lower proportion of the LR patients had cirrhosis (51.4% vs 71.8%, *P < *0.001).

### Performance of the nomogram in predicting MVI risks

Among 905 patients who underwent LR, 257 (28.4%) were identified as having MVI on post-operative histopathological examination. The C-index of the nomogram for predicting MVI was 0.809. Based on a nomogram cut-off value of 200 points, the sensitivity and specificity were 70.8% and 90.8%, respectively. The positive and negative predictive values for differentiating the presence from the absence of MVI were 75.5% and 88.7%, respectively. Of 905 patients with LR, 241 (26.6%) and 664 (73.4%) were predicted to have a high and low risk of MVI, respectively, through the nomogram. Among them, 182 (182/241, 75.5%) and 75 (75/664, 11.3%) patients had MVI on post-operative histopathology. Among 117 LT patients, 41 (35.0%) had histopathologically confirmed MVI. The C-index of the nomogram for predicting MVI was 0.838. Based on the same nomogram cut-off value, the predictive sensitivity, specificity, and positive and negative predictive values were 78.0%, 89.4%, 80.0%, and 88.3%, respectively. Among the 117 patients, 40 (34.2%) and 77 (65.8%) were categorized as high- and low-risk MVI groups, respectively, with 32 (32/40, 80.0%) and 9 (9/77, 11.7%) having MVI on histopathology, respectively.

### Survival outcomes of patients undergoing LR and LT based on predicted MVI risks

The median follow-up period was 47.6 (range, 3.0–86.8) months for the LR patients and 73.6 (3.3–185.4) months for the LT patients, respectively.

**Figure 1.**
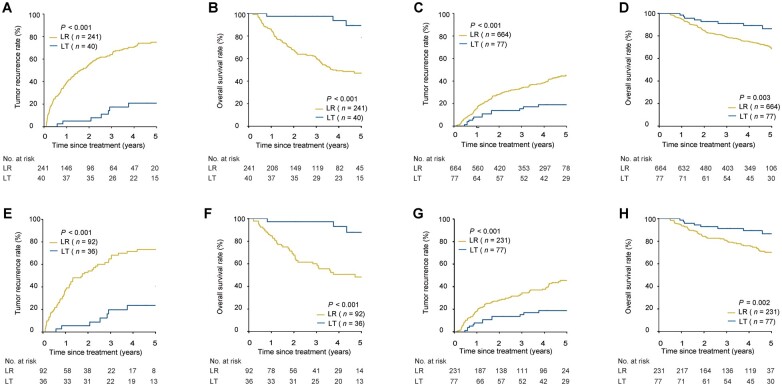
Tumor recurrence and OS for patients undergoing LR vs LT based on predicted MVI risks before or after PSM. (A) Recurrence for patients with high risk of MVI before PSM. (B) OS for patients with high risk of MVI before PSM. (C) Recurrence for patients with low risk of MVI before PSM. (D) OS for patients with low risk of MVI before PSM. (E) Recurrence for patients with high risk of MVI after PSM. (F) OS for patients with high risk of MVI after PSM. (G) Recurrence for patients with low risk of MVI after PSM. (H) OS for patients with low risk of MVI after PSM. OS, overall survival; LR, liver resection; LT, liver transplantation; MVI, microvascular invasion; PSM, propensity score matching.

**Figure 2.**
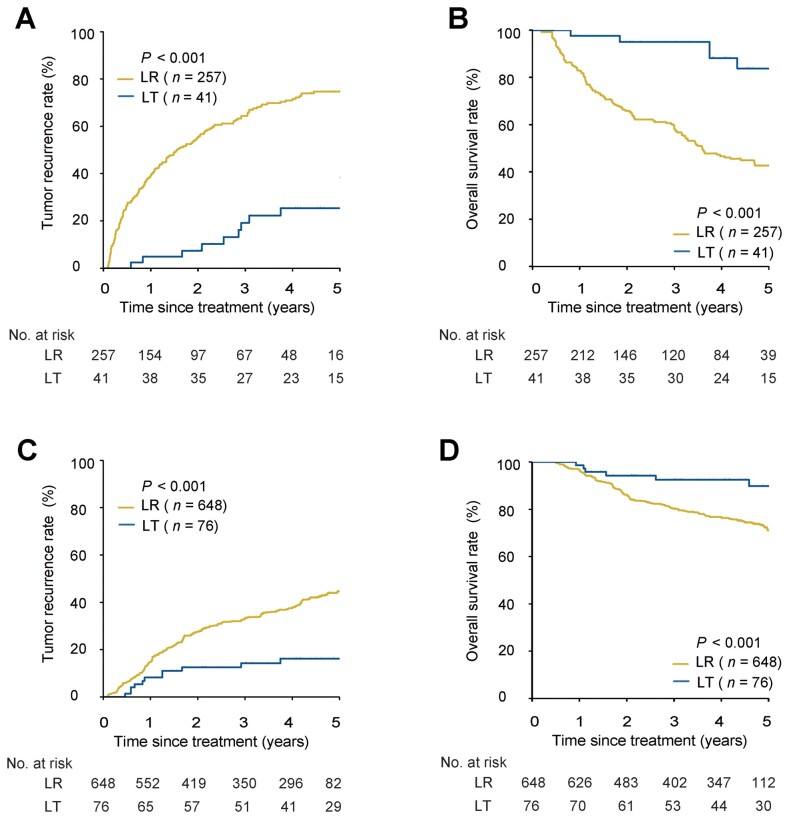
Tumor recurrence and OS for patients undergoing LR vs LT based on the presence or absence of MVI on post-operative histopathology before PSM. (A) Recurrence in patients with MVI. (B) OS in patients with MVI. (C) Recurrence in patients without MVI. (D) OS in patients without MVI. OS, overall survival; LR, liver resection; LT, liver transplantation; MVI, microvascular invasion; PSM, propensity score matching.

In this cohort and before PSM, patients undergoing LR showed worse survival outcomes than patients undergoing LT in those with a high or low risk of MVI. The 1-, 3-, and 5-year recurrence and OS rates in the LR vs LT groups were 39.0%, 63.9%, and 74.9% vs 5.0%, 17.4%, and 20.8%, respectively, and 85.9%, 60.8%, and 47.0% vs 97.5%, 97.5%, and 89.3%, respectively, in high-risk MVI patients (both *P *<* *0.001); the corresponding recurrence and OS rates were 15.4%, 34.1%, and 45.0% vs 8.1%, 15.4%, and 19.0%, respectively (*P *<* *0.001), and 94.9%, 78.9%, and 68.7% vs 98.6%, 91.1%, and 86.5%, respectively (*P *=* *0.003) in low-risk MVI patients (Figure 1A–D). Similar results were obtained in the presence and absence of MVI, as demonstrated by using post-operative histopathology. The 1-, 3-, and 5-year recurrence and OS rates of patients in the LR and LT groups were 39.1%, 64.4%, and 74.7% vs 4.9%, 19.2%, and 25.4%, respectively, and 82.9%, 58.7%, and 42.7% vs 97.6%, 95.0%, and 83.8%, respectively (both *P *<* *0.001), for patients with MVI on histopathology; the corresponding recurrence and OS rates were 14.8%, 33.4%, and 44.5% vs 8.2%, 14.2%, and 16.1%, respectively (*P *<* *0.001), and 96.3%, 80.1%, and 71.1% vs 98.6%, 92.5%, and 89.8%, respectively (*P *<* *0.001) for patients without MVI (Figure 2A–D). Multivariable analysis demonstrated that LT was a protective factor for recurrence and OS compared with LR in patients with a high risk of MVI (recurrence: hazard ratio [HR], 0.19; 95% confidence interval [CI], 0.09–0.36. OS: HR, 0.10; 95% CI, 0.03–0.28) or a low risk of MVI (recurrence: HR, 0.37; 95% CI, 0.21–0.65. OS: HR, 0.34; 95% CI, 0.16–0.70) (Table 2 and Supplementary Table 2).

**Table 1.** Baseline characteristics of patients with HCC within the Milan criteria based on predicted MVI risk who underwent LR or LT

Variable	Before PSM						After PSM					
	High-risk MVI			Low-risk MVI			**High-risk MVI** ^e^			**Low-risk MVI** ^f^		
	LR	LT	*P*	LR	LT	*P*	LR	LT	*P*	LR	LT	*P*
	(*n *=* *241)	(*n *=* *40)		(*n *=* *664)	(*n *=* *77)		(*n *=* *92)	(*n *=* *36)		(*n *=* *231)	(*n *=* *77)	
**Age**, ≤65 years	222 (92.1)	36 (90.0)	0.754	608 (91.6)	68 (88.3)	0.458	85 (92.4)	32 (88.9)	0.502	210 (90.9)	68 (88.3)	0.657
**Gender**, male	197 (81.7)	34 (85.0)	0.783	562 (84.6)	68 (88.3)	0.493	77 (83.7)	31 (86.1)	0.946	199 (86.1)	68 (88.3)	0.771
**HBeAg**, positive	92 (38.2)	7 (17.5)	0.018	236 (35.5)	18 (23.4)	0.045	13 (14.1)	7 (19.4)	0.636	57 (24.7)	18 (23.4)	0.939
**HBV-DNA level**, ≥2,000 IU/mL	177 (73.4)	27 (67.5)	0.556	296 (44.6)	33 (42.9)	0.868	63 (68.5)	24 (66.7)	0.907	94 (40.7)	33 (42.9)	0.841
**TBIL**, <1.0 mg/dL	212 (87.9)	29 (72.5)	0.018	498 (75.0)	53 (68.8)	0.300	72 (78.3)	28 (77.8)	0.887	169 (73.2)	53 (68.8)	0.557
**ALB**, ≥3.5 g/dL	231 (95.9)	34 (85.0)	0.015	643 (96.8)	69 (89.6)	0.007	88 (95.7)	33 (91.7)	0.401	221 (95.7)	72 (93.5)	0.540
**INR**, <1.2	228 (94.6)	36 (90.0)	0.278	641 (96.5)	72 (93.5)	0.199	90 (97.8)	33 (91.7)	0.135	225 (97.4)	72 (93.5)	0.150
**PLT**, ≥100 × 10^9^/L	197 (81.7)	25 (62.5)	0.011	558 (84.0)	57 (74.0)	0.040	78 (84.8)	27 (75.0)	0.298	184 (79.7)	62 (80.5)	0.869
**AFP**, >400 µg/L	128 (53.1)	21 (52.5)	0.943	125 (18.8)	10 (13.0)	0.217	45 (48.9)	18 (50.0)	0.881	42 (18.2)	10 (13.0)	0.380
**CEA**, >5 µmol/L	18 (7.47)	4 (10.0)	0.532	49 (7.38)	6 (7.79)	0.896	10 (10.9)	3 (8.33)	0.960	21 (9.09)	6 (7.79)	0.907
**CA19-9**, >37 U/L	54 (22.4)	9 (22.5)	0.866	144 (21.7)	15 (19.5)	0.764	17 (18.5)	8 (22.2)	0.816	54 (23.4)	15 (19.5)	0.581
**Child–Pugh grade,** A	238 (98.8)	36 (90.0)	0.009	648 (97.6)	67 (87.0)	<0.001	91 (98.9)	35 (97.2)	0.485	215 (93.1)	67 (87.0)	0.156
**Cirrhosis** (I)^b^, yes	123 (51.0)	31 (77.5)	0.003	342 (51.5)	53 (68.8)	0.006	62 (67.4)	28 (77.8)	0.347	165 (71.4)	53 (68.8)	0.772
**Tumor diameter** (I)^a^, cm	3.5 ± 1.0	3.3 ± 1.0	0.112	2.7 ± 1.0	2.6 ± 1.1	0.267	3.5 ± 1.1	3.3 ± 0.9	0.297	2.8 ± 1.0	2.7 ± 1.1	0.434
**Tumor number** (I), solitary	170 (70.5)	14 (35.0)	<0.001	616 (92.8)	59 (76.6)	<0.001	41 (44.6)	13 (36.1)	0.502	194 (84.0)	59 (76.6)	0.198
**Tumor capsule** (I)^c^, incomplete	201 (83.4)	30 (75.0)	0.288	216 (32.5)	25 (32.5)	0.991	74 (80.4)	27 (75.0)	0.662	72 (31.2)	25 (32.5)	0.944
**Typical dynamic pattern** ^d^, presence	208 (86.3)	32 (80.0)	0.421	367 (55.3)	49 (63.6)	0.201	73 (79.3)	29 (80.6)	0.853	120 (51.9)	49 (63.6)	0.098
**Cirrhosis** (P)^b^, yes	127 (52.7)	30 (75.0)	0.014	362 (54.5)	52 (67.5)	0.040	62 (67.4)	27 (75.0)	0.530	169 (73.2)	52 (67.5)	0.422
**Tumor diameter** (P)^a^, cm	3.4 ± 1.0	3.1 ± 1.1	0.936	2.7 ± 1.0	2.6 ± 1.1	0.224	3.4 ± 1.1	3.2 ± 0.9	0.185	2.7 ± 1.0	2.6 ± 1.1	0.653
**Tumor number** (P), solitary	174 (72.2)	17 (42.5)	<0.001	608 (91.6)	58 (75.3)	<0.001	49 (53.3)	16 (44.4)	0.484	187 (81.0)	58 (75.3)	0.370
**Tumor capsule** (P), incomplete	201 (83.4)	31 (77.5)	0.493	225 (33.9)	27 (35.1)	0.936	74 (80.4)	27 (75.0)	0.662	81 (35.1)	27 (35.1)	0.914
**Surgical margin**, cm, ≥1	102 (42.3)	–	–	265 (39.9)	–	–	37 (40.2)	–	–	90 (38.9)	–	–
**Edmondson–Steiner grade**, I/II	174 (72.2)	26 (65.0)	0.458	332 (50.0)	35 (45.5)	0.526	68 (73.9)	23 (63.9)	0.364	115 (49.8)	35 (45.5)	0.599
**MVI**, presence	182 (75.5)	32 (80.0)	0.678	75 (11.3)	9 (11.7)	0.918	67 (72.8)	31 (86.1)	0.173	28 (12.1)	9 (11.7)	0.934

LR, liver resection; LT, liver transplantation; MVI, microvascular invasion; PSM, propensity score matching; HBeAg, hepatitis B e antigen; HBV-DNA, hepatitis B virus-deoxyribonucleic acid; TBIL, total bilirubin; ALB, albumin; INR, international normalized ratio; PLT, platelet; AFP, alpha fetoprotein; CEA, carcinoembryonic antigen; CA 19–9, carbohydrate antigen 19–9.

a Values are presented as mean (standard deviation); other values are presented as number of patients followed by percentage in parentheses.

b (I) was based on preoperative imaging studies; (P) was based on post-operative pathological examinations.

c If one nodule had incomplete capsule among multiple nodules, it was defined as incomplete capsule.

d Presence of both arterial enhancement and washout on contrast-enhanced MRI.

e The preoperative variables used in the PSM were HBeAg, TBIL, ALB, PLT, Child–Pugh grade, tumor number, and cirrhosis.

f The preoperative variables used in the PSM were Child–Pugh grade, HBeAg, ALB, PLT, Child–Pugh grade, tumor number, and cirrhosis.

**Table 2.** Multivariable Cox regression analysis of recurrence and OS in patients with high- or low-risk MVI

Variable	High-risk MVI				Low-risk MVI			
	Recurrence		OS		Recurrence		OS	
	HR (95% CI)	*P*	HR (95% CI)	*P*	HR (95% CI)	*P*	HR (95% CI)	*P*
***Before PSM***								
**HBV-DNA level**, ≥2,000 vs <2,000 IU/mL	2.05 (1.41–2.99)	<0.001	2.13 (1.34–3.39)	0.001	2.02 (1.59–2.58)	<0.001	1.96 (1.45–2.65)	<0.001
**ALB**, ≥3.5 vs <3.5 g/dL							0.35 (0.18–0.66)	0.001
**AFP**, >400 vs ≤400 µg/L	1.73 (1.27–2.37)	<0.001	1.94 (1.34–2.82)	0.001	–	–	–	–
**Tumor diameter** (I)^a^, cm	–	–	–	–	1.51 (1.35–1.71)	<0.001	1.39 (1.20–1.60)	<0.001
**Tumor number** (I), multiple vs solitary	–	–	–	–	2.10 (1.42–3.09)	<0.001	2.01 (1.26–3.21)	0.003
**Tumor capsule** (I), incomplete vs complete	–	–	–	–	1.71 (1.33–2.21)	<0.001	1.80 (1.32–2.45)	<0.001
**Type of treatment**, LT vs LR	0.19 (0.09–0.36)	<0.001	0.10 (0.03–0.28)	<0.001	0.37 (0.21–0.65)	<0.001	0.34 (0.16–0.70)	0.003
***After PSM***								
**HBV-DNA level**, 2,000 ≥ vs <2,000 IU/mL	2.41 (1.38–4.22)	0.002	2.88 (1.35–6.13)	0.006	2.07 (1.41–3.04)	<0.001	2.07 (1.28–3.35)	0.003
**Child–Pugh grade**, B7 vs A					1.77 (1.00–3.13)	0.048		
**AFP**, >400 vs ≤400 µg/L	1.71 (1.02–2.89)	0.041	2.35 (1.19–4.65)	0.014	–	–	–	–
**Tumor diameter** (I), cm					1.37 (1.14–1.65)	0.001	0.29 (1.03–1.62)	0.024
**Tumor number** (I)^a^, multiple vs solitary	–	–	–	–	2.33 (1.46–3.71)	<0.001	2.23 (1.28–3.88)	0.005
**Tumor capsule** (I), incomplete vs complete					1.58 (1.05–2.36)	0.026	1.74 (1.07–2.82)	0.024
**Type of treatment**, LT vs LR	0.18 (0.09–0.37)	<0.001	0.12 (0.04–0.37)	<0.001	0.37 (0.21–0.66)	0.001	0.36 (0.17–0.78)	0.009

OS, overall survival; MVI, microvascular invasion; PSM, propensity score matching; HBV-DNA, hepatitis B virus-deoxyribonucleic acid; AFP, alpha fetoprotein; LT, liver transplantation; LR, liver resection; HR, hazard ratio; CI, confidence interval.

a (I) was based on preoperative imaging studies.

After PSM, the baseline characteristics between the LR and LT groups were well balanced in patients with a high risk (92 vs 36) or a low risk (231 vs 77) of MVI (Table 1). The 1-, 3-, and 5-year recurrence and OS rates in the LR vs LT groups were 37.0%, 64.8%, and 73.2% vs 5.6%, 19.8%, and 23.6%, respectively, and 84.8%, 59.9%, and 48.1% vs 97.2%, 97.2%, and 87.8%, respectively (both *P *<* *0.001), for high-risk MVI patients; the corresponding recurrence and OS rates were 18.3%, 34.8%, and 45.7% vs 8.1%, 15.4%, and 19.0%, respectively (*P *<* *0.001), and 93.5%, 80.2%, and 70.0% vs 98.6%, 91.1%, and 86.5%, respectively (*P *=* *0.002) for low-risk MVI patients (Figure 1E–H). Compared with LR, LT was associated with a reduced risk of recurrence and OS in the patients with a high risk of MVI (recurrence: HR, 0.18; 95% CI, 0.09–0.37. OS: HR, 0.12; 95% CI, 0.04–0.37) or a low risk of MVI (recurrence: HR, 0.37; 95% CI, 0.21–0.66. OS: HR, 0.36; 95% CI, 0.17–0.78) (Table 2 and Supplementary Table 3).

### Survival outcomes of patients undergoing AR and LT based on predicted MVI risks

Among 524 patients who underwent LR with AR, 145 (27.7%) showed MVI on post-operative histopathological examination. Based on the MVI prediction, 131 (25.0%) and 393 (75.0%) patients were defined as having a high or low risk of MVI, respectively.

**Figure 3.**
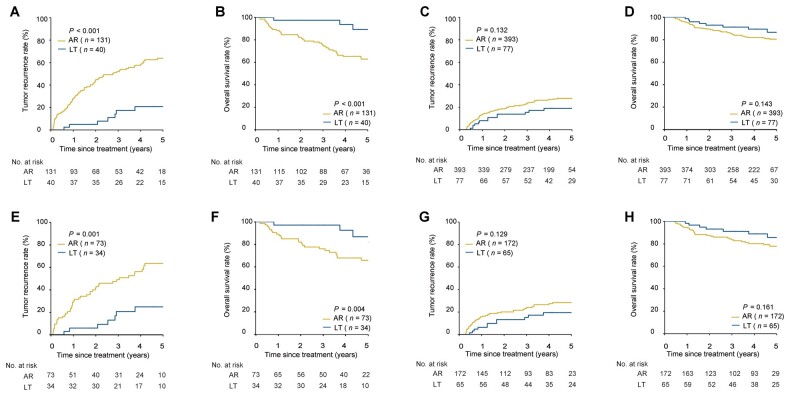
Tumor recurrence and OS for patients undergoing AR vs LT based on predicted risks for MVI before or after PSM. (A) Recurrence for patients with high risk for MVI before PSM. (B) OS for patients with high risk of MVI before PSM. (C) Recurrence for patients with low risk of MVI before PSM. (D) OS for patients with low risk of MVI before PSM. (E) Recurrence for patients with high risk of MVI after PSM. (F) OS for patients with high risk of MVI after PSM. (G) Recurrence for patients with low risk of MVI after PSM. (H) OS for patients with low risk of MVI after PSM. OS, overall survival; AR, anatomical resection; LT, liver transplantation; MVI, microvascular invasion; PSM, propensity score matching.

**Figure 4.**
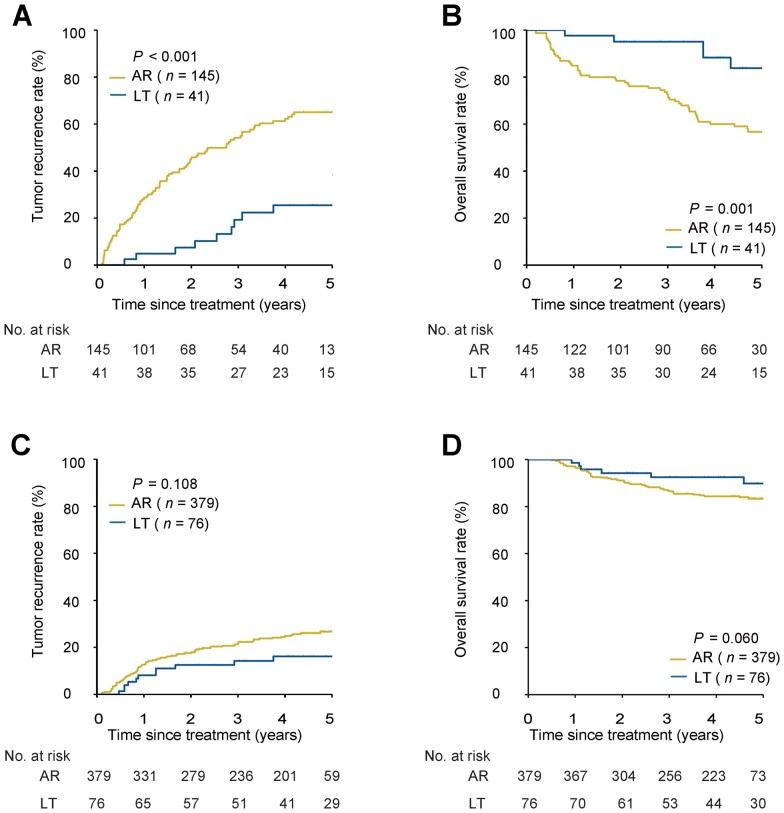
Tumor recurrence and OS for patients undergoing AR vs LT based on the presence or absence of MVI on post-operative histopathology before PSM. (A) Recurrence for patients with MVI. (B) OS for patients with MVI. (C) Recurrence for patients without MVI. (D) OS for patients without MVI. OS, overall survival; AR, anatomical resection; LT, liver transplantation; MVI, microvascular invasion; PSM, propensity score matching.

Before PSM, the baseline features of AR and LT patients were unbalanced in both high- and low-risk MVI patients (Table 3). Among high-risk MVI patients, survival outcomes of patients undergoing AR were worse than those undergoing LT. There were no significant differences in prognosis between the patients receiving AR and LT in low-risk MVI patients. The 1-, 3-, and 5-year recurrence and OS rates in high-risk MVI patients undergoing AR vs LT were 28.3%, 52.0%, and 63.9% vs 5.0%, 17.4%, and 20.8%, respectively, and 88.5%, 75.6%, and 62.9% vs 97.5%, 97.5%, and 89.3%, respectively (both *P *<* *0.001); the corresponding recurrence and OS rates for low-risk MVI patients were 13.3%, 23.9%, and 27.8% vs 8.1%, 15.4%, and 19.0%, respectively (*P *=* *0.132), and 94.9%, 84.9%, and 80.3% vs 98.6%, 91.1%, and 86.5%, respectively (*P *=* *0.143) (Figure 3A–D). These results were further confirmed using the presence or absence of MVI on post-operative histopathology. The 1-, 3-, and 5-year recurrence and OS rates of patients in the AR vs LT groups were 28.6%, 54.1%, and 65.0% vs 4.9%, 19.2%, and 25.4%, respectively (*P *<* *0.001), and 84.8%, 72.0%, and 56.6% vs 97.6%, 95.0%, and 83.8%, respectively (*P *=* *0.001), for patients who had MVI; the corresponding recurrence and OS rates were 12.7%, 22.3%, and 26.7% vs 8.2%, 14.2%, and 16.1%, respectively (*P *=* *0.108), and 96.6%, 85.3%, and 83.3% vs 98.6%, 92.5%, and 89.8%, respectively (*P *=* *0.060), for patients who had no MVI (Figure 4A–D). LT was the protective factor for recurrence and OS in patients with a high risk of MVI (recurrence: HR, 0.23; 95% CI, 0.11–0.46. OS: HR, 0.11; 95% CI, 0.04–0.34) (Table 4 and Supplementary Table 4).

**Table 3.** Baseline characteristics of patients with HCC within the Milan criteria based on predicted MVI risk who underwent LR or LT

Variable	Before PSM						After PSM					
	High-risk MVI			Low-risk MVI			**High-risk MVI** ^e^			**Low-risk MVI** ^f^		
	AR	LT	*P*	AR	LT	*P*	AR	LT	*P*	AR	LT	*P*
	(*n *=* *131)	(*n *=* *40)		(*n *=* *393)	(*n *=* *77)		(*n *=* *73)	(*n *=* *34)		(*n *=* *172)	(*n *=* *65)	
**Age**, ≤65 years	119 (90.8)	36 (90.0)	0.873	363 (92.4)	68 (88.3)	0.340	69 (94.5)	30 (88.2)	0.261	159 (92.4)	57 (87.7)	0.373
**Gender**, male	115 (87.8)	34 (85.0)	0.849	324 (82.4)	68 (88.3)	0.272	64 (87.7)	30 (88.2)	0.980	139 (80.8)	56 (86.2)	0.441
**HBeAg**, positive	44 (33.6)	7 (17.5)	0.080	148 (37.7)	18 (23.4)	0.023	18 (24.7)	6 (17.6)	0.575	46 (26.7)	16 (24.6)	0.867
**HBV-DNA level**, ≥2,000 IU/mL	90 (68.7)	27 (67.5)	0.678	168 (42.7)	33 (42.9)	0.986	50 (68.5)	22 (64.7)	0.867	77 (44.8)	28 (43.1)	0.931
**TBIL**, <1.0 mg/dL	115 (87.7)	29 (72.5)	0.038	306 (77.9)	53 (68.8)	0.119	58 (79.5)	25 (73.5)	0.664	122 (70.9)	45 (69.2)	0.923
**ALB**, ≥3.5 g/dL	124 (94.7)	34 (85.0)	0.094	379 (96.4)	69 (89.6)	0.022	68 (93.2)	31 (91.2)	0.707	166 (96.5)	61 (93.8)	0.468
**INR**, <1.2	124 (94.7)	36 (90.0)	0.286	375 (95.4)	72 (93.5)	0.560	66 (90.4)	30 (88.2)	0.740	162 (94.2)	60 (92.3)	0.562
**PLT**, ≥100 × 10^9^/L	105 (80.2)	25 (62.5)	0.038	328 (83.5)	57 (74.0)	0.071	58 (79.5)	25 (73.5)	0.664	138 (80.2)	51 (78.5)	0.903
**AFP**, >400 µg/L	40 (30.5)	21 (52.5)	0.019	74 (18.8)	10 (13.0)	0.289	32 (43.8)	15 (44.1)	0.864	35 (20.3)	8 (12.3)	0.213
**CEA**, >5 µmol/L	7 (5.34)	4 (10.0)	0.293	28 (7.12)	6 (7.79)	0.836	5 (6.85)	3 (8.82)	0.707	12 (6.98)	5 (7.69)	0.785
**CA19-9**, >37 U/L	30 (22.9)	9 (22.5)	0.953	99 (25.2)	15 (19.5)	0.256	20 (27.4)	8 (23.5)	0.851	49 (28.5)	13 (20.0)	0.246
**Child–Pugh grade,** A	129 (98.5)	36 (90.0)	0.027	384 (97.7)	67 (87.0)	<0.001	72 (98.6)	32 (94.1)	0.236	163 (94.8)	60 (92.3)	0.538
**Cirrhosis** (I)^b^, yes	66 (50.4)	31 (77.5)	0.004	213 (54.2)	53 (68.8)	0.025	50 (68.5)	26 (76.5)	0.536	111 (64.5)	41 (63.1)	0.955
**Tumor diameter** (I)^a^, cm	3.5 ± 0.9	3.2 ± 1.0	0.052	2.2 ± 0.6	2.7 ± 1.1	<0.001	3.5 ± 1.0	3.4 ± 1.0	0.604	2.3 ± 0.6	2.5 ± 1.0	0.126
**Tumor number** (I), solitary	88 (67.2)	14 (35.0)	0.001	355 (90.3)	59 (76.6)	0.001	37 (50.7)	13 (38.2)	0.320	145 (84.3)	50 (76.9)	0.256
**Tumor capsule** (I)^c^, incomplete	110 (84.0)	30 (75.0)	0.292	144 (36.6)	25 (32.5)	0.570	59 (80.8)	25 (73.5)	0.547	64 (37.2)	22 (33.8)	0.742
**Typical dynamic pattern** ^d^, presence	115 (87.8)	32 (80.0)	0.327	229 (58.3)	49 (63.6)	0.454	62 (84.9)	27 (79.4)	0.665	90 (52.3)	41 (63.1)	0.181
**Cirrhosis** (P)^b^, yes	67 (51.1)	30 (75.0)	0.013	220 (56.0)	52 (67.5)	0.080	50 (68.5)	26 (76.5)	0.536	115 (66.9)	40 (61.5)	0.538
**Tumor diameter** (P)^a^, cm	3.5 ± 0.97	3.2 ± 1.03	0.078	2.0 ± 0.6	2.6 ± 1.0	<0.001	3.2 ± 1.0	3.1 ± 1.0	0.790	2.2 ± 0.6	2.3 ± 1.0	0.211
**Tumor number** (P), solitary	88 (67.2)	17 (42.5)	0.009	349 (88.8)	58 (75.3)	0.003	37 (50.7)	15 (44.1)	0.671	147 (85.5)	50 (76.9)	0.170
**Tumor capsule** (P), incomplete	110 (84.0)	31 (77.5)	0.418	149 (37.9)	27 (35.1)	0.731	59 (80.8)	26 (76.5)	0.794	69 (40.1)	23 (35.4)	0.605
**Surgical margin**, cm, ≥1	68 (51.9)	–	–	201 (51.1)	–	–	39 (53.4)	–	–	87 (50.5)	–	–
**Edmondson–Steiner grade**, I/II	88 (67.2)	26 (65.0)	0.949	181 (46.1)	35 (45.5)	0.923	47 (64.4)	23 (67.6)	0.911	80 (46.5)	29 (44.6)	0.908
**MVI**, presence	96 (73.3)	32 (80.0)	0.516	49 (12.5)	9 (11.7)	0.999	51 (69.9)	28 (82.4)	0.257	22 (12.8)	9 (13.8)	0.879

AR, anatomical resection; LT, liver transplantation; MVI, microvascular invasion; PSM, propensity score matching; HBeAg, hepatitis B e antigen; HBV-DNA, hepatitis B virus-deoxyribonucleic acid; TBIL, total bilirubin; ALB, albumin; INR, international normalized ratio; PLT, platelet; AFP, alpha fetoprotein; CEA, carcinoembryonic antigen; CA 19–9, carbohydrate antigen 19–9.

a Values are presented as mean (standard deviation); other values are presented as number of patients followed by percentage in parentheses.

b (I) was based on preoperative imaging studies; (P) was based on post-operative pathological examinations.

c If one nodule had incomplete capsule among multiple nodules, it was defined as incomplete capsule.

d Presence of both arterial enhancement and washout on contrast-enhanced MRI.

e The preoperative variables used in the PSM were TBIL, PLT, AFP, Child–Pugh grade, cirrhosis, and tumor number.

f The preoperative variables used in the PSM were HBeAg, ALB, Child–Pugh grade, cirrhosis tumor diameter, and tumor number.

**Table 4.** Multivariable Cox regression analysis of recurrence and OS between patients with high- or low-risk MVI undergoing AR and LT

Variable	High-risk MVI				Low-risk MVI			
	Recurrence		OS		Recurrence		OS	
	HR (95% CI)	*P*	HR (95% CI)	*P*	HR (95% CI)	*P*	HR (95% CI)	*P*
***Before PSM***								
**HBV-DNA level**, ≥2,000 vs <2,000 IU/mL	2.10 (1.27–3.65)	0.002	2.45 (1.21–4.97)	0.013	1.97 (1.37–2.87)	<0.001	2.22 (1.40–3.52)	0.001
**ALB**, ≥3.5 vs <3.5 g/dL	–	–	–	–	0.40 (0.19–0.81)	0.012	0.34 (0.15–0.77)	0.011
**Child–Pugh grade**, B7 vs A					2.09 (1.07–4.08)	0.031		
**Tumor number** (I)^a^, multiple vs solitary	–	–	–	–	2.02 (1.25–3.27)	0.004	2.16 (1.22–3.84)	0.008
**Tumor capsule** (I), incomplete vs complete	–	–	–	–	2.09 (1.07–4.08)	<0.001	2.31 (1.42–3.77)	0.001
**Type of treatment**, LT vs AR	0.23 (0.11–0.46)	<0.001	0.11 (0.04–0.34)	<0.001	–	–	–	–
***After PSM***								
**HBV-DNA level**, ≥2,000 vs <2,000 IU/mL	1.94 (1.02–3.68)	0.041	3.63 (1.38–9.55)	0.009	2.28 (1.33–3.90)	0.003	2.60 (1.35–5.01)	0.004
**Tumor number** (I)^a^, multiple vs solitary	2.58 (1.38–4.82)	0.003	–	–	3.08 (1.62–5.88)	0.001	3.29 (1.50–7.19)	0.003
**Tumor capsule** (I), incomplete vs complete	–	–	–	–	2.60 (1.47–4.58)	0.001	3.33 (1.65–6.71)	0.001
**Type of treatment**, LT vs AR	0.24 (0.11–0.53)	<0.001	0.17 (0.06–0.52)	0.002	–	–	–	–

OS, overall survival; MVI, microvascular invasion; PSM, propensity score matching; HBV-DNA, hepatitis B virus-deoxyribonucleic acid; LT, liver transplantation; AR, anatomical resection; HR, hazard ratio; CI, confidence interval.

a (I) was based on preoperative imaging studies.

After PSM, well-balanced baselines were achieved between the AR and LT groups in patients with a high risk (73 vs 34) and low risk (172 vs 65) of MVI (Table 3). The 1-, 3-, and 5-year recurrence and OS rates in the AR vs LT groups among patients with a high risk of MVI were 30.1%, 49.1%, and 63.5% vs 5.9%, 20.6%, and 24.8%, respectively (*P *=* *0.001), and 89.0%,76.0%, and 65.7% vs 97.1%, 97.1%, and 86.7%, respectively (*P *=* *0.004); the corresponding recurrence and OS rates among patients with a low risk of MVI were 15.7%, 24.0%, and 28.3% vs 6.4%, 15.2%, and 19.4%, respectively (*P *=* *0.129), and 94.2%, 83.5%, and 77.8% vs 98.3%, 91.1%, and 85.7%, respectively (*P *=* *0.161) (Figure 3E–H). On multivariable analysis, LT was associated with a decreased risk of recurrence and OS but only in the patients with a high risk of MVI (recurrence: HR, 0.24; 95% CI, 0.11–0.53. OS: HR, 0.17; 95% CI, 0.06–0.52) (Table 4 and Supplementary Table 5).

## Discussion

This study demonstrated the superiority of LT over LR in terms of long-term survival and recurrence in patients with HCC within the Milan criteria and were predicted to have a high or low risk of MVI. After dividing the cohort into resection types, LT was still associated with a better prognosis than AR in patients with a high risk of MVI. However, there was no significant difference in survival outcomes between LT and AR groups among patients with a low risk of MVI. The results based on the predicted MVI risks were confirmed using the presence or absence of MVI, as demonstrated by using post-operative histopathology.

Using predicted MVI risks in preoperative decision-making is a novel strategy and challenge in HCC surgery because MVI can only be diagnosed by using post-operative histopathology [2, 3, 18]. The reported methods for the preoperative prediction of MVI include several typical radiological features [20], serum or tumor biomarkers [19], genomic sequencing data [36], radiogenomic venous invasion [21], an artificial neural network that initially proposed the usefulness of a clinicopathological model in the preoperative prediction of MVI [22], and a nomogram with the advantages of good sensitivity and specificity in predicting MVI [23]. In this study, the nomogram used to predict MVI performed well with C-indices of 0.809 and 0.838 for LR and LT patients, respectively. Based on a nomogram cut-off value of 200 points, the sensitivity, specificity, and positive and negative predictive values for predicting MVI were good. Furthermore, histopathologically confirmed MVI status and predicted MVI risks showed similar impacts on the prognoses of patients undergoing either LR or LT, suggesting the potential of the model in making preoperative decisions regarding treatment.

Our study demonstrated that LT had a better oncological effect than LR based on either the status of MVI on histopathology or the predicted risks of MVI obtained from the nomogram in patients with HCC within the Milan criteria. Previous studies comparing the prognosis for patients undergoing LT and LR did not consider the type of LR [6, 8–10, 13], which may have affected the comparative results. In this study, although AR might decrease the chance of recurrence by removing the entire related vasculature and hepatic territory [28], its survival benefits were still fewer than those of LT in patients at high risk of MVI. A previous study by our team and international colleagues showed that the survival benefits for patients undergoing LT for resectable HCC did not improve compared with those for patients undergoing LR. However, 32% of the included patients were beyond the Milan criteria and the types of LR were not analysed separately in that study [12]. The present study suggests that the ability of LT to eliminate MVI was remarkably stronger than that of AR. Among patients with a low risk of MVI, LT seemed to provide better survival outcomes for patients than AR, though the prognostic difference did not reach statistical significance. This result could be interpreted as the elimination of both the tumor load and the underlying cirrhosis in LT patients. However, AR can provide favorable survival benefits over NAR for early-stage HCC patients possibly through more effective eradication of pre-existing small nodules or micrometastasis from the primary tumors [37, 38].

Ablation is also commonly used to treat early-stage HCC. The results of comparing survival outcomes of patients undergoing ablation and LR have remained inconsistent in previous studies [4]. Some studies have suggested that ablation has a similar therapeutic effect in HCC of ≤2 cm but a decreased effect in HCC of >3 cm compared with LR [39, 40]. Our previous study demonstrated that LR with either AR or NAR was better than ablation in terms of long-term survival outcomes in patients with early-stage HCC with a predicted high risk of MVI [41]. From the perspective of oncology and combined with data from previous reports and the present study, LT, followed by LR and ablation, provides the best curative effect in patients with early-stage HCC who are at a high risk of MVI. The effects of LR, especially AR, may not be inferior to those of LT and may be better than those of ablation in patients with a low risk of MVI.

Our study has several limitations. First, this study did not include patients who received salvage LT, although salvage LT has become an important strategy to prolong long-term survival [42, 43]. Our study did not further compare the prognosis for patients undergoing AR and NAR because this issue based on the presence or absence of MVI has been reported many times [37, 44]. Second, although eligibility criteria and PSM analysis were used to adjust for the baseline characteristics of LR and LT patients, selection bias could not be completely eliminated. Third, this study focused mainly on oncological effects. In addition to the estimated preoperative MVI risks, conventional factors that influence preoperative decisions, such as tumor location, FLR volume, degree of cirrhosis, and technical feasibility, were not further analysed. Notably, several studies have suggested the positive association between cirrhosis and the incidence of MVI. Although cirrhosis was not included as a variable in the nomogram to predict MVI, its exact effect on the formation of MVI and underlying mechanism needs to be further investigated. Fourth, the results of the current study may only apply to patients with HBV-related HCC, although the adverse impact of MVI on surgical prognosis has also been demonstrated in HCV-infected HCC [45]. Finally, more sensitive, specific, and applicable tools must be developed to improve the preoperative prediction of MVI.

In conclusion, patients with HCC within the Milan criteria who were predicted to have a high or low risk of MVI benefited more from LT than LR when the two approaches were selected as first-line treatments. AR provided survival benefits that were not significantly different from LT for patients with a low risk of MVI. Our study suggests the potential role of the predicted risks of MVI in preoperative treatment decision-making between LR and LT in patients with early-stage HCC.

## Supplementary Material

goad035_Supplementary_Data
