# Supplementary material for: Liver resection versus liver transplantation for hepatocellular carcinoma within the Milan criteria based on estimated microvascular invasion risks
Source: Gastroenterol Rep (Oxf). 2023 Jun 26;11:goad035. doi: 10.1093/gastro/goad035 (PMC10293589; doi:10.1093/gastro/goad035)
Supplement: goad035_Supplementary_Data [file goad035_supplementary_data.zip › 2022-429 Supplementary Table 1-5.docx]

**Supplementary Table 1. Baseline characteristics patients in the whole population who underwent LR or LT**

| **Variable** | **Number (%)/Value (mean ± SD)** | | ***P*^*^** |
| --- | --- | --- | --- |
|  | **LR (*n* = 905)** | **LT (*n* = 117)** |  |
| **Age**, years |  |  | 0.306 |
| ≤65 | 830 (91.7) | 104 (88.9) |  |
| >65 | 75 (8.3) | 13 (11.1) |  |
| **Gender** |  |  | 0.355 |
| Male | 759 (83.9) | 102 (87.2) |  |
| Female | 146 (16.1) | 15 (12.8) |  |
| **HBeAg** |  |  | 0.001 |
| Negative | 577 (63.8) | 92 (78.7) |  |
| Positive | 328 (36.2) | 25 (21.3) |  |
| **HBV-DNA level**, IU/mL |  |  | 0.919 |
| <2,000 | 432 (47.7) | 57 (48.7) |  |
| ≥2,000 | 473 (52.3) | 60 (51.3) |  |
| **TBIL**, mg/dL |  |  | 0.055 |
| <1.0 | 710 (78.5) | 82 (70.1) |  |
| ≥1.0 | 195 (21.5) | 35 (29.9) |  |
| **ALB**, g/dL |  |  | <0.001 |
| <3.5 | 31 (3.43) | 14(12.0) |  |
| ≥3.5 | 874 (96.6) | 103 (88.0) |  |
| **INR** |  |  | 0.109 |
| <1.2 | 869 (96.0) | 108 (92.3) |  |
| ≥1.2 | 36 (3.98) | 9 (7.69) |  |
| **PLT**, ×10^9^/L |  |  | 0.001 |
| <100 | 150 (16.6) | 35 (29.9) |  |
| ≥100 | 755 (83.4) | 82 (70.1) |  |
| **AFP**, µg/L |  |  | 0.824 |
| ≤400 | 652 (72.0) | 86 (73.5) |  |
| >400 | 253 (28.0) | 31 (26.5) |  |
| **CEA**, µmol/L |  |  | 0.659 |
| ≤5 | 838 (92.6) | 107 (91.5) |  |
| >5 | 67 (7.4) | 10 (8.5) |  |
| **CA19-9**, U/L |  |  | 0.827 |
| ≤37 | 707 (78.1) | 93 (79.5) |  |
| >37 | 198 (21.9) | 24 (20.5) |  |
| **Child-Pugh grade** |  |  | <0.001 |
| A | 886 (97.9) | 103 (88.0) |  |
| B7 | 19 (2.1) | 14 (12.0) |  |
| **Cirrhosis** (I)^†^ |  |  | <0.001 |
| No | 440 (48.6) | 33 (28.2) |  |
| Yes | 465 (51.4) | 84 (71.8) |  |
| **Tumor diameter** (I), cm | 3.0±1.0 | 2.9±1.1 | 0.115 |
| **Tumor number** (I) |  |  | <0.001 |
| Solitary | 786 (86.9) | 73 (62.4) |  |
| Multiple | 119 (13.1) | 44 (37.6) |  |
| **Tumor capsule** (I)^‡^ |  |  | 0.927 |
| Incomplete | 417 (46.1) | 55 (47.0) |  |
| Complete | 488 (53.9) | 62 (53.0) |  |
| **Typical dynamic pattern**^§^ |  |  | 0.268 |
| Absence | 330 (36.5) | 36 (30.8) |  |
| Presence | 575 (63.5) | 81 (69.2) |  |
| **Cirrhosis** (P)^†^ |  |  | 0.001 |
| No | 416 (46.0) | 35 (29.9) |  |
| Yes | 489 (54.0) | 82 (70.1) |  |
| **Tumor diameter** (P), cm | 2.9±1.0 | 2.8±1.1 | 0.125 |
| **Tumor number** (P) |  |  | <0.001 |
| Solitary | 782 (86.4) | 75 (64.1) |  |
| Multiple | 123 (13.6) | 42 (35.9) |  |
| **Tumor capsule** (P)^‡^ |  |  | 0.618 |
| Incomplete | 426 (47.1) | 58 (49.6) |  |
| Complete | 479 (52.9) | 59 (50.4) |  |
| **Edmondson-Steiner grade** |  |  | 0.500 |
| I/II | 506 (55.9) | 61 (52.1) |  |
| III/IV | 399 (44.1) | 56 (47.9) |  |
| **MVI risk** (predicted) |  |  | 0.107 |
| Low | 664 (73.4) | 77 (65.8) |  |
| High | 241 (26.6) | 40 (35.2) |  |
| **MVI** (P) |  |  | 0.168 |
| Absence |  |  |  |
| Presence |  |  |  |

**Abbreviations:** LR, liver resection; LT, liver transplantation; HBeAg, hepatitis B e antigen; HBV-DNA, hepatitis B virus-deoxyribonucleic acid; TBIL, total bilirubin; ALB, albumin; INR, international normalized ratio; PLT, platelet; AFP, alpha fetoprotein; CEA, carcinoembryonic antigen; CA 19-9, carbohydrate antigen 19-9; MVI, microvascular invasion.

^†^: (I): imaging studies; (P): postoperative pathological examinations.

^‡^: if any one nodule had incomplete capsule among multiple nodules, it was defined as incomplete capsule.

^§^: presence of both arterial enhancement and washout on contrast-enhanced MRI.

^*^: continuous variables were compared with the paired t test or Mann-Whitney U test, as

appropriate; categorical variables were compared with the chi-square test or Fisher’s exact

test.

**Supplementary Table 2. Univariable Cox regression analysis of recurrence and OS in patients with high- or low-risk MVI before PSM**

| **Variable** | **High-risk MVI** | | | | |  | **Low-risk MVI** | | | | |
| --- | --- | --- | --- | --- | --- | --- | --- | --- | --- | --- | --- |
|  | **Recurrence** | |  | **OS** | |  | **Recurrence** | |  | **OS** | |
|  | **HR (95% CI)** | ***P*** |  | **HR (95% CI)** | ***P*** |  | **HR (95% CI)** | ***P*** |  | **HR (95% CI)** | ***P*** |
| **Age**, >65 vs ≤65 years | 0.57 (0.31-1.02) | 0.058 |  | 0.46 (0.21-1.00) | 0.051 |  | 1.17 (0.79-1.71) | 0.432 |  | 1.43 (0.92-2.24) | 0.115 |
| **Gender**, female vs male | 0.95 (0.64-1.42) | 0.818 |  | 1.37 (0.89-2.12) | 0.157 |  | 1.09 (0.79-1.51) | 0.592 |  | 1.10 (0.73-1.65) | 0.653 |
| **HBeAg**, positive vs negative | 1.52 (1.12-2.05) | 0.006 |  | 1.52 (1.07-2.17) | 0.020 |  | 1.14 (0.89-1.45) | 0.308 |  | 1.21 (0.90-1.64) | 0.212 |
| **HBV-DNA level**, ≥2,000 vs <2,000 IU/mL | 1.82 (1.26-2.61) | 0.001 |  | 1.85 (1.19-2.89) | 0.006 |  | 2.00 (1.58-2.54) | <0.001 |  | 2.01 (1.49-2.71) | <0.001 |
| **TBIL**, ≥1.0 vs <1.0 mg/dL | 0.89 (0.58-1.36) | 0.586 |  | 0.97 (0.60-1.58) | 0.913 |  | 1.33 (1.03-1.72) | 0.030 |  | 1.33 (0.97-1.84) | 0.077 |
| **ALB**, ≥3.5 vs <3.5 g/dL | 1.61 (0.79-3.27) | 0.191 |  | 1.76 (0.72-4.30) | 0.218 |  | 0.59 (0.35-1.00) | 0.048 |  | 0.54 (0.29-1.00) | 0.049 |
| **INR**, ≥1.2 vs <1.2 | 0.74 (0.39-1.41) | 0.362 |  | 0.68 (0.31-1.46) | 0.320 |  | 1.02 (0.53-1.99) | 0.947 |  | 1.26 (0.59-2.68) | 0.555 |
| **PLT**, ≥100 vs <100 ×10^9^/L | 1.26 (0.86-1.84) | 0.245 |  | 1.35 (0.85-2.13) | 0.202 |  | 1.01 (0.74-1.37) | 0.966 |  | 1.12 (0.75-1.65) | 0.580 |
| **AFP**, >400 vs ≤400 µg/L | 1.54 (1.15-2.08) | 0.004 |  | 1.74 (1.22-2.48) | 0.002 |  | 0.94 (0.69-1.28) | 0.705 |  | 0.90 (0.61-1.35) | 0.622 |
| **CEA**, >5 vs ≤5 µmol/L | 1.41 (0.86-2.30) | 0.171 |  | 1.18 (0.63-2.19) | 0.607 |  | 1.23 (0.81-1.87) | 0.326 |  | 1.22 (0.72-2.07) | 0.459 |
| **CA19-9**, >37 vs ≤37 U/L | 0.90 (0.63-1.28) | 0.560 |  | 1.04 (0.70-1.55) | 0.857 |  | 0.88 (0.65-1.18) | 0.381 |  | 0.97 (0.68-1.38) | 0.848 |
| **Child-Pugh grade**, B7 vs A | 0.40 (0.13-1.25) | 0.115 |  | 0.54 (0.17-1.71) | 0.291 |  | 1.61 (0.94-2.75) | 0.085 |  | 1.33 (0.65-2.71) | 0.440 |
| **Cirrhosis** (I)^†^, yes vs no | 0.92 (0.69-1.24) | 0.591 |  | 1.08 (0.76-1.53) | 0.664 |  | 0.79 (0.62-0.99) | 0.044 |  | 0.86 (0.64-1.16) | 0.329 |
| **Tumor diameter** (I), cm | 1.09 (0.94-1.26) | 0.238 |  | 1.04 (0.88-1.23) | 0.643 |  | 1.44 (1.29-1.61) | <0.001 |  | 1.32 (1.15-1.51) | <0.001 |
| **Tumor number** (I), multiple vs solitary | 0.78 (0.57-1.06) | 0.118 |  | 0.64 (0.43-0.93) | 0.020 |  | 1.49 (1.03-2.17) | 0.035 |  | 1.44 (0.91-2.28) | 0.115 |
| **Tumor capsule** (I), incomplete vs complete | 1.06 (0.72-1.54) | 0.770 |  | 1.28 (0.80-2.04) | 0.307 |  | 1.38 (1.08-1.75) | 0.009 |  | 1.47 (1.09-1.98) | 0.012 |
| **Typical dynamic pattern**, presence vs absence | 1.11 (0.72-1.70) | 0.643 |  | 1.64 (0.93-2.91) | 0.090 |  | 0.68 (0.53-0.86) | 0.001 |  | 0.77 (0.58-1.04) | 0.093 |
| **Type of treatment**, LT vs LR | 0.22 (0.12-0.40) | <0.001 |  | 0.11 (0.04-0.31) | <0.001 |  | 0.40 (0.24-0.69) | 0.001 |  | 0.37 (0.18-0.74) | 0.005 |

**Abbreviation**s: OS, overall survival; MVI, microvascular invasion; PSM, propensity score matching; HBeAg, hepatitis B e antigen; HBV-DNA, hepatitis B virus-deoxyribonucleic acid; TBIL, total bilirubin; ALB, albumin; INR, international normalized ratio; PLT, platelet; AFP, alpha fetoprotein; CEA, carcinoembryonic antigen; CA 19-9, carbohydrate antigen 19-9; LT, liver transplantation; LR, liver resection; hazard ratio; CI, confidence interval.

^†^: (I) was based on preoperative imaging studies.

**Supplementary Table 3. Univariable Cox regression analysis of recurrence and OS in patients with high- or low-risk MVI after PSM**

| **Variable** | **High-risk MVI** | | | | |  | **Low-risk MVI** | | | | |
| --- | --- | --- | --- | --- | --- | --- | --- | --- | --- | --- | --- |
|  | **Recurrence** | |  | **OS** | |  | **Recurrence** | |  | **OS** | |
|  | **HR (95% CI)** | ***P*** |  | **HR (95% CI)** | ***P*** |  | **HR (95% CI)** | ***P*** |  | **HR (95% CI)** | ***P*** |
| **Age**, >65 vs ≤65 years | 0.37 (0.13-1.03) | 0.057 |  | 0.16 (0.02-1.18) | 0.073 |  | 0.93 (0.48-1.78) | 0.822 |  | 0.98 (0.45-2.15) | 0.966 |
| **Gender**, female vs male | 0.71 (0.34-1.49) | 0.365 |  | 1.34 (0.63-2.89) | 0.447 |  | 0.94 (0.52-1.67) | 0.822 |  | 0.75 (0.34-1.63) | 0.465 |
| **HBeAg**, positive vs negative | 1.10 (0.59-2.05) | 0.756 |  | 1.30 (0.61-2.79) | 0.500 |  | 1.01 (0.64-1.58) | 0.976 |  | 0.97 (0.56-1.70) | 0.928 |
| **HBV-DNA level**, ≥2,000 vs <2,000 IU/mL | 1.98 (1.15-3.41) | 0.014 |  | 2.34 (1.13-4.84) | 0.022 |  | 2.26 (1.54-3.31) | <0.001 |  | 2.37 (1.47-3.81) | <0.001 |
| **TBIL**, ≥1.0 vs <1.0 mg/dL | 1.28 (0.74-2.21) | 0.373 |  | 1.22 (0.64-2.35) | 0.547 |  | 1.46 (0.97-2.18) | 0.067 |  | 1.37 (0.83-2.26) | 0.213 |
| **ALB**, ≥3.5 vs <3.5 g/dL | 1.71 (0.62-4.69) | 0.297 |  | 2.20 (0.53-9.08) | 0.276 |  | 0.57 (0.29-1.14) | 0.112 |  | 0.51 (0.23-1.12) | 0.093 |
| **INR**, ≥1.2 vs <1.2 | 0.18 (0.02-1.29) | 0.087 |  | 0.29 (0.04-2.19) | 0.232 |  | 0.53 (0.13-2.13) | 0.368 |  | 0.98 (0.24-4.00) | 0.976 |
| **PLT**, ≥100 vs <100 ×10^9^/L | 1.13 (0.63-2.02) | 0.686 |  | 1.62 (0.73-3.62) | 0.238 |  | 1.13 (0.69-1.83) | 0.626 |  | 1.29 (0.69-2.40) | 0.427 |
| **AFP**, >400 vs ≤400 µg/L | 1.66 (1.04-2.63) | 0.032 |  | 2.32 (1.28-4.18) | 0.005 |  | 1.26 (0.78-2.03) | 0.346 |  | 1.34 (0.74-2.42) | 0.328 |
| **CEA**, >5 vs ≤5 µmol/L | 2.03 (1.06-3.89) | 0.034 |  | 1.46 (0.62-3.46) | 0.391 |  | 0.66 (0.31-1.41) | 0.282 |  | 0.58 (0.21-1.59) | 0.291 |
| **CA19-9**, >37 vs ≤37 U/L | 0.68 (0.37-1.27) | 0.226 |  | 0.71 (0.33-1.53) | 0.384 |  | 0.95 (0.61-1.50) | 0.838 |  | 1.14 (0.67-1.96) | 0.622 |
| **Child-Pugh grade**, B7 vs A | 0.49 (0.07-3.61) | 0.487 |  | 0.74 (0.10-5.50) | 0.767 |  | 1.76 (1.00-3.10) | 0.048 |  | 1.43 (0.68-3.01) | 0.341 |
| **Cirrhosis** (I)^†^, yes vs no | 1.67 (0.97-2.87) | 0.065 |  | 2.37 (1.11-5.06) | 0.026 |  | 0.80 (0.53-1.20) | 0.283 |  | 0.80 (0.48-1.31) | 0.371 |
| **Tumor diameter** (I), cm | 1.24 (0.98-1.58) | 0.078 |  | 1.02 (0.77-1.36) | 0.889 |  | 1.28 (1.08-1.52) | 0.004 |  | 1.20 (0.97-1.47) | 0.091 |
| **Tumor number** (I), multiple vs solitary | 0.85 (0.53-1.34) | 0.480 |  | 0.78 (0.44-1.38) | 0.397 |  | 1.86 (1.20-2.88) | 0.005 |  | 1.87 (1.10-3.18) | 0.020 |
| **Tumor capsule** (I), incomplete vs complete | 1.42 (0.79-2.54) | 0.238 |  | 1.85 (0.83-4.14) | 0.132 |  | 1.60 (1.09-2.35) | 0.017 |  | 1.64 (1.02-2.64) | 0.041 |
| **Typical dynamic pattern**, presence vs absence | 0.89 (0.51-1.58) | 0.699 |  | 1.54 (0.69-3.44) | 0.290 |  | 0.58 (0.39-0.85) | 0.005 |  | 0.68 (0.43-1.10) | 0.115 |
| **Type of treatment**, LT vs LR | 0.24 (0.12-0.46) | <0.001 |  | 0.17 (0.06-0.48) | 0.001 |  | 0.37 (0.21-0.65) | 0.001 |  | 0.33 (0.16-0.71) | 0.004 |

**Abbreviation**s: OS, overall survival; MVI, microvascular invasion; PSM, propensity score matching; HBeAg, hepatitis B e antigen; HBV-DNA, hepatitis B virus-deoxyribonucleic acid; TBIL, total bilirubin; ALB, albumin; INR, international normalized ratio; PLT, platelet; AFP, alpha fetoprotein; CEA, carcinoembryonic antigen; CA 19-9, carbohydrate antigen 19-9; LT, liver transplantation; LR, liver resection; hazard ratio; CI, confidence interval.

^†^: (I) was based on preoperative imaging studies.

**Supplementary Table 4. Univariable Cox regression analysis of recurrence and OS between AR and LT groups among patients with high- or low-risk MVI before PSM**

| **Variable** | **High-risk MVI** | | | | |  | **Low-risk MVI** | | | | |
| --- | --- | --- | --- | --- | --- | --- | --- | --- | --- | --- | --- |
|  | **Recurrence** | |  | **OS** | |  | **Recurrence** | |  | **OS** | |
|  | **HR (95% CI)** | ***P*** |  | **HR (95% CI)** | ***P*** |  | **HR (95% CI)** | ***P*** |  | **HR (95% CI)** | ***P*** |
| **Age**, >65 vs ≤65 years | 0.50 (0.22-1.16) | 0.106 |  | 0.34 (0.11-1.11) | 0.074 |  | 1.65 (0.96-2.84) | 0.071 |  | 1.95 (1.05-3.62) | 0.034 |
| **Gender**, female vs male | 0.64 (0.32-1.28) | 0.211 |  | 0.76 (0.32-1.76) | 0.519 |  | 1.35 (0.85-2.12) | 0.203 |  | 1.52 (0.89-2.61) | 0.126 |
| **HBeAg**, positive vs negative | 1.52 (0.98-2.35) | 0.059 |  | 1.73 (1.01-2.97) | 0.047 |  | 1.04 (0.71-1.52) | 0.851 |  | 1.01 (0.63-1.62) | 0.958 |
| **HBV-DNA level**, ≥2,000 vs <2,000 IU/mL | 1.87 (1.14-3.08) | 0.013 |  | 2.13 (1.08-4.22) | 0.030 |  | 2.01 (1.39-2.91) | <0.001 |  | 2.32 (1.47-3.65) | <0.001 |
| **TBIL**, ≥1.0 vs <1.0 mg/dL | 0.94 (0.53-1.67) | 0.837 |  | 1.12 (0.58-2.16) | 0.730 |  | 1.42 (0.95-2.12) | 0.089 |  | 1.55 (0.96-2.50) | 0.075 |
| **ALB**, ≥3.5 vs <3.5 g/dL | 1.77 (0.72-4.36) | 0.216 |  | 2.6 (0.63-10.67) | 0.185 |  | 0.45 (0.23-0.86) | 0.015 |  | 0.40 (0.19-0.84) | 0.015 |
| **INR**, ≥1.2 vs <1.2 | 0.50 (0.18-1.36) | 0.174 |  | 0.35 (0.08-1.45) | 0.148 |  | 1.00 (0.41-2.44) | 0.995 |  | 1.64 (0.66-4.06) | 0.286 |
| **PLT**, ≥100 vs <100 ×10^9^/L | 1.09 (0.66-1.78) | 0.747 |  | 1.07 (0.57-1.98) | 0.841 |  | 1.07 (0.66-1.74) | 0.777 |  | 1.25 (0.68-2.31) | 0.477 |
| **AFP**, >400 vs ≤400 µg/L | 0.90 (0.58-1.38) | 0.625 |  | 0.84 (0.49-1.45) | 0.534 |  | 1.23 (0.78-1.93) | 0.372 |  | 1.56 (0.93-2.62) | 0.093 |
| **CEA**, >5 vs ≤5 µmol/L | 1.41 (0.65-3.05) | 0.388 |  | 0.91 (0.28-2.92) | 0.870 |  | 1.07 (0.54-2.10) | 0.854 |  | 1.24 (0.57-2.71) | 0.582 |
| **CA19-9**, >37 vs ≤37 U/L | 0.77 (0.46-1.29) | 0.319 |  | 0.86 (0.46-1.59) | 0.623 |  | 1.09 (0.72-1.65) | 0.681 |  | 1.32 (0.81-2.13) | 0.262 |
| **Child-Pugh grade**, B7 vs A | 0.37 (0.09-1.52) | 0.168 |  | 0.52 (0.12-2.16) | 0.366 |  | 2.57 (1.34-4.93) | 0.005 |  | 1.98 (0.85-4.62) | 0.116 |
| **Cirrhosis** (I)^†^, yes vs no | 0.96 (0.63-1.45) | 0.835 |  | 1.23 (0.73-2.09) | 0.440 |  | 0.78 (0.54-1.12) | 0.184 |  | 1.07 (0.68-1.67) | 0.777 |
| **Tumor diameter** (I), cm | 1.17 (0.95-1.44) | 0.149 |  | 1.09 (0.85-1.41) | 0.501 |  | 1.06 (0.84-1.34) | 0.637 |  | 1.02 (0.77-1.36) | 0.887 |
| **Tumor number** (I), multiple vs solitary | 0.95 (0.63-1.44) | 0.813 |  | 0.74 (0.43-1.27) | 0.276 |  | 2.04 (1.28-3.25) | 0.003 |  | 2.09 (1.20-3.64) | 0.009 |
| **Tumor capsule** (I), incomplete vs complete | 0.98 (0.59-1.65) | 0.953 |  | 0.94 (0.50-1.77) | 0.844 |  | 1.83 (1.27-2.63) | 0.001 |  | 1.85 (1.19-2.89) | 0.006 |
| **Typical dynamic pattern**, presence vs absence | 1.41 (0.73-2.72) | 0.306 |  | 3.20 (1.0-10.24) | 0.050 |  | 0.67 (0.46-0.96) | 0.031 |  | 0.84 (0.53-1.32) | 0.441 |
| **Type of treatment**, LT vs LR | 0.30 (0.16-0.57) | <0.001 |  | 0.15 (0.05-0.42) | <0.001 |  | 0.65 (0.37-1.14) | 0.132 |  | 0.58 (0.28-1.20) | 0.143 |

**Abbreviation**s: OS, overall survival; MVI, microvascular invasion; PSM, propensity score matching; HBeAg, hepatitis B e antigen; HBV-DNA, hepatitis B virus-deoxyribonucleic acid; TBIL, total bilirubin; ALB, albumin; INR, international normalized ratio; PLT, platelet; AFP, alpha fetoprotein; CEA, carcinoembryonic antigen; CA 19-9, carbohydrate antigen 19-9; LT, liver transplantation; AR, anatomical resection; hazard ratio; CI, confidence interval.

^†^: (I) was based on preoperative imaging studies.

**Supplementary Table 5. Univariable Cox regression analysis of recurrence and OS between AR and LT groups in patients with high- or low-risk MVI after PSM**

| **Variable** | **High-risk MVI** | | | | |  | **Low-risk MVI** | | | | |
| --- | --- | --- | --- | --- | --- | --- | --- | --- | --- | --- | --- |
|  | **Recurrence** | |  | **OS** | |  | **Recurrence** | |  | **OS** | |
|  | **HR (95% CI)** | ***P*** |  | **HR (95% CI)** | ***P*** |  | **HR (95% CI)** | ***P*** |  | **HR (95% CI)** | ***P*** |
| **Age**, >65 vs ≤65 years | 0.37 (0.13-1.03) | 0.057 |  | 0.17 (0.02-1.31) | 0.089 |  | 1.25 (0.56-2.76) | 0.584 |  | 1.25 (0.49-3.21) | 0.640 |
| **Gender**, female vs male | 0.71 (0.34-1.49) | 0.365 |  | 0.84 (0.25-2.75) | 0.769 |  | 1.51 (0.81-2.80) | 0.192 |  | 1.44 (0.69-3.02) | 0.333 |
| **HBeAg**, positive vs negative | 1.10 (0.59-2.05) | 0.756 |  | 1.88 (0.91-3.89) | 0.089 |  | 1.20 (0.67-2.13) | 0.538 |  | 1.01 (0.49-2.06) | 0.982 |
| **HBV-DNA level**, ≥2,000 vs <2,000 IU/mL | 1.98 (1.15-3.41) | 0.014 |  | 2.76 (1.07-7.16) | 0.036 |  | 2.37 (1.39-4.02) | 0.001 |  | 2.74 (1.44-5.19) | 0.002 |
| **TBIL**, ≥1.0 vs <1.0 mg/dL | 1.28 (0.74-2.21) | 0.373 |  | 1.55 (0.74-3.24) | 0.248 |  | 1.73 (1.01-2.94) | 0.044 |  | 1.98 (1.06-3.69) | 0.032 |
| **ALB**, ≥3.5 vs <3.5 g/dL | 1.71 (0.62-4.69) | 0.297 |  | 1.73 (0.41-7.24) | 0.454 |  | 0.43 (0.19-1.02) | 0.055 |  | 0.55 (0.19-1.58) | 0.267 |
| **INR**, ≥1.2 vs <1.2 | 0.18 (0.02-1.29) | 0.087 |  | 0.42 (0.10-1.77) | 0.237 |  | 0.98 (0.31-3.14) | 0.973 |  | 1.57 (0.48-5.10) | 0.454 |
| **PLT**, ≥100 vs <100 ×10^9^/L | 1.13 (0.63-2.02) | 0.686 |  | 2.83 (1.00-8.06) | 0.051 |  | 1.10 (0.57-2.13) | 0.769 |  | 1.31 (0.58-2.95) | 0.521 |
| **AFP**, >400 vs ≤400 µg/L | 1.66 (1.04-2.63) | 0.032 |  | 0.95 (0.48-1.87) | 0.876 |  | 1.21 (0.64-2.30) | 0.553 |  | 1.33 (0.63-2.81) | 0.451 |
| **CEA**, >5 vs ≤5 µmol/L | 2.03 (1.06-3.89) | 0.034 |  | 1.61 (0.48-5.38) | 0.436 |  | 0.92 (0.33-2.54) | 0.874 |  | 0.98 (0.30-3.17) | 0.971 |
| **CA19-9**, >37 vs ≤37 U/L | 0.68 (0.37-1.27) | 0.226 |  | 0.40 (0.15-1.04) | 0.06 |  | 1.57 (0.92-2.68) | 0.099 |  | 1.96 (1.05-3.65) | 0.034 |
| **Child-Pugh grade**, B7 vs A | 0.49 (0.07-3.61) | 0.487 |  | 0.70 (0.09-5.21) | 0.725 |  | 3.43 (1.68-6.99) | 0.001 |  | 3.01 (1.26-7.21) | 0.013 |
| **Cirrhosis** (I)^†^, yes vs no | 1.67 (0.97-2.87) | 0.065 |  | 1.52 (0.69-3.37) | 0.303 |  | 0.84 (0.50-1.43) | 0.530 |  | 0.93 (0.50-1.76) | 0.832 |
| **Tumor diameter** (I), cm | 1.24 (0.98-1.58) | 0.078 |  | 1.01 (0.73-1.40) | 0.937 |  | 1.17 (0.86-1.61) | 0.321 |  | 1.13 (0.77-1.64) | 0.530 |
| **Tumor number** (I), multiple vs solitary | 0.85 (0.53-1.34) | 0.480 |  | 1.35 (0.68-2.69) | 0.389 |  | 2.07 (1.16-3.69) | 0.014 |  | 1.95 (0.98-3.91) | 0.058 |
| **Tumor capsule** (I), incomplete vs complete | 1.42 (0.79-2.54) | 0.238 |  | 1.14 (0.49-2.61) | 0.764 |  | 1.91 (1.14-3.21) | 0.014 |  | 2.07 (1.11-3.83) | 0.021 |
| **Typical dynamic pattern**, presence vs absence | 0.89 (0.51-1.58) | 0.699 |  | 0.73 (0.52-1.01) | 0.051 |  | 0.83 (0.50-1.40) | 0.491 |  | 0.99 (0.53-1.84) | 0.963 |
| **Type of treatment**, LT vs LR | 0.24 (0.12-0.46) | <0.001 |  | 0.21 (0.07-0.61) | 0.004 |  | 0.60 (0.32-1.16) | 0.129 |  | 0.56 (0.25-1.26) | 0.161 |

**Abbreviations**: OS, overall survival; MVI, microvascular invasion; PSM, propensity score matching; HBeAg, hepatitis B e antigen; HBV-DNA, hepatitis B virus-deoxyribonucleic acid; TBIL, total bilirubin; ALB, albumin; INR, international normalized ratio; PLT, platelet; AFP, alpha fetoprotein; CEA, carcinoembryonic antigen; CA 19-9, carbohydrate antigen 19-9; LT, liver transplantation; AR, anatomical resection; hazard ratio; CI, confidence interval.

^†^: (I) was based on preoperative imaging studies.
