# Supplementary figures and images for: Liver resection versus liver transplantation for hepatocellular carcinoma within the Milan criteria based on estimated microvascular invasion risks
Source: Gastroenterol Rep (Oxf). 2023 Jun 26;11:goad035. doi: 10.1093/gastro/goad035 (PMC10293589; doi:10.1093/gastro/goad035)

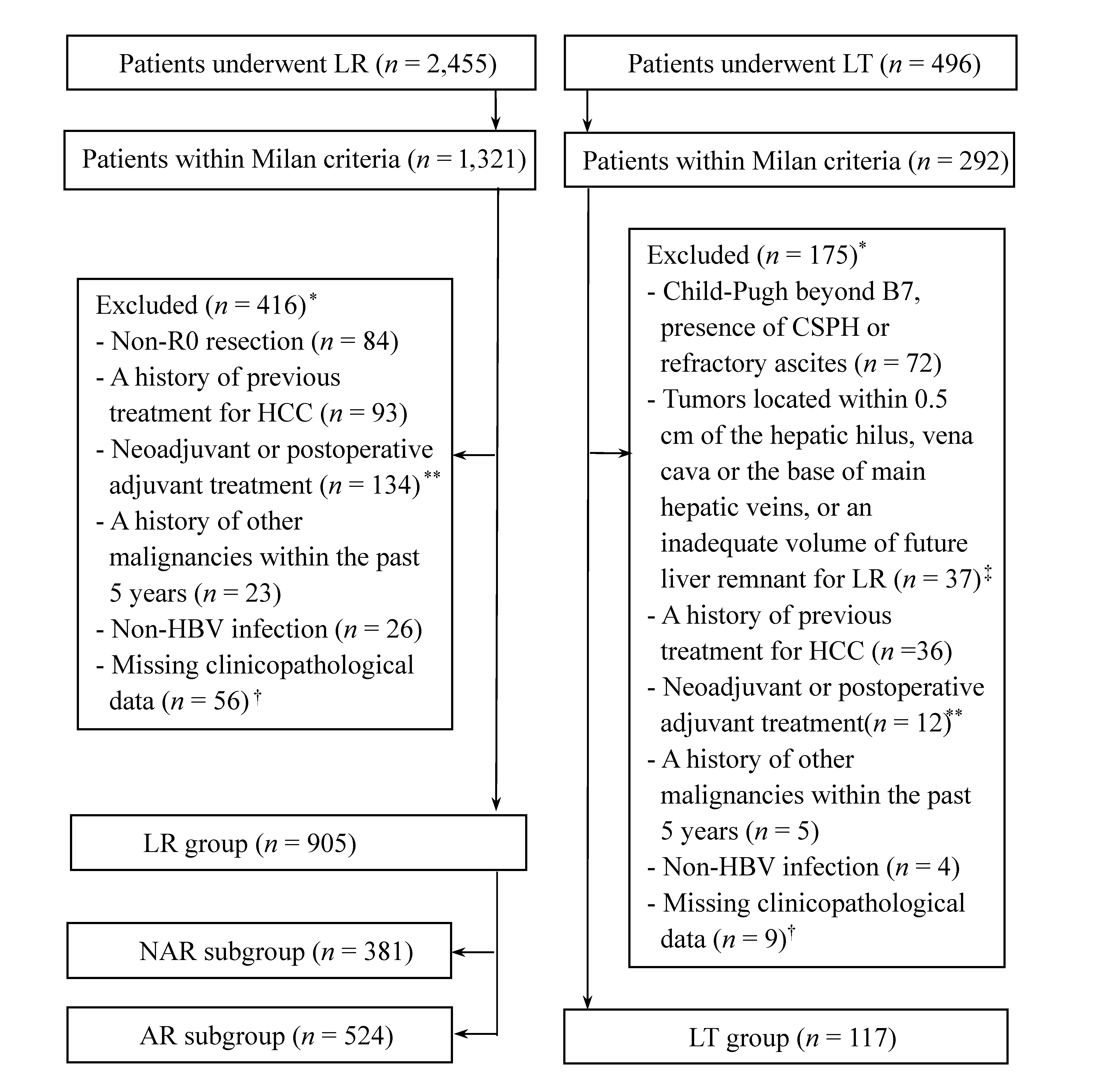

Supplement: goad035_Supplementary_Data [file goad035_supplementary_data.zip › 2022-429 Supplementary Figure 1.tif]
